# Asymmetric author-topic model for knowledge discovering of big data in toxicogenomics

**DOI:** 10.3389/fphar.2015.00081

**Published:** 2015-04-20

**Authors:** Ming-Hua Chung, Yuping Wang, Hailin Tang, Wen Zou, John Basinger, Xiaowei Xu, Weida Tong

**Affiliations:** ^1^Department of Mathematical Sciences, University of ArkansasFayetteville, AR, USA; ^2^Division of Bioinformatics and Biostatistics, National Center for Toxicological Research, US Food and Drug AdministrationJefferson, AR, USA; ^3^Department of Information Science, University of Arkansas at Little RockLittle Rock, AR, USA

**Keywords:** toxicogenomics, machine learning, probabilistic topic modeling, author-topic model, bioinformatics, TG-GATEs

## Abstract

The advancement of high-throughput screening technologies facilitates the generation of massive amount of biological data, a big data phenomena in biomedical science. Yet, researchers still heavily rely on keyword search and/or literature review to navigate the databases and analyses are often done in rather small-scale. As a result, the rich information of a database has not been fully utilized, particularly for the information embedded in the interactive nature between data points that are largely ignored and buried. For the past 10 years, probabilistic topic modeling has been recognized as an effective machine learning algorithm to annotate the hidden thematic structure of massive collection of documents. The analogy between text corpus and large-scale genomic data enables the application of text mining tools, like probabilistic topic models, to explore hidden patterns of genomic data and to the extension of altered biological functions. In this paper, we developed a generalized probabilistic topic model to analyze a toxicogenomics dataset that consists of a large number of gene expression data from the rat livers treated with drugs in multiple dose and time-points. We discovered the hidden patterns in gene expression associated with the effect of doses and time-points of treatment. Finally, we illustrated the ability of our model to identify the evidence of potential reduction of animal use.

## Introduction

As first introduced in 1999, toxicogenomics has emerged as a new subdiscipline of toxicology to take advantage of the newly available genomics profiling technique to gain an enhanced understanding of toxicity at the molecular level (Schena et al., 1995; Derisi et al., 1996; Nuwaysir et al., 1999). Since then, toxicogenomics significantly contributes to toxicological research and has provided an avenue for joining of multidisciplinary sciences including engineering and informatics into traditional toxicological research (Afshari et al., 2011). On the other hand, due to high computational cost and lack of advanced knowledge discovery as well as data mining tools, the pace of toxicogenomics has been tardy in recent years (Chen et al., 2012). First, a significant deterrent has been the enormous size of toxicogenomic datasets. With perhaps thousands of samples and tens of thousands of genes, the tremendous size of the toxicogenomic database often is cumbersome to handle, analyze and interpret. Gene selection (i.e., selecting relevant genes) and grouping genes (i.e., dealing only partial data at a time) has often been used to reduce complexity and make analyses more tractable (Rogers et al., 2005). However, both gene selection and grouping run the risk of losing valuable information contained in excluded data. Hence, a method that can efficiently handle the entire data without losing potentially valuable information is desirable. Second, any given biological phenomenon normally involves multiple biological pathways and mechanisms. Currently, some existing clustering algorithms like hierarchical cluster analysis and k-means only allow individuals to be assigned into mutually exclusive clusters. To capture the reality of biological phenomena in gene expression data, we need an algorithm to assign individuals into multiple clusters and to give each cluster a summary of most important genes. One might argue that some fuzzy clustering algorithms (Pal and Bezdek, 1995; Fu and Medico, 2007) are able to assign multiple clusters, yet very few existing algorithm provide much interpretability for clusters. In order to thoroughly utilize the rich interaction in a large database, we desire to organize our samples into meaningful clusters which can be directly linked by actual biological pathways.

The introduction of *Latent Dirichlet Allocation* (LDA) (Blei et al., 2003) along with its predecessor *Probabilistic Latent Semantic Analysis* (Hofmann, 1999) provide a new type of statistical models, namely, probabilistic topic models that have become a standard approach to analyze large collections of unstructured text documents. For a large corpus, probabilistic topic models assume the existence of latent variables (i.e., topics) that govern the likelihood of appearance for each word. Topics are defined as distributions over a fixed vocabulary. Based on the most likely words in each topic, we are able to interpret the meanings of topics. This intuition can be seamlessly transformed into genomics datasets. For a large toxicogenomic data, we assume that there exist latent biological processes that govern alteration of gene expression levels after samples are treated with drugs at various dose levels and time-points. Each latent biological process is characterized by a distribution of a fixed number of genes. By annotating the mostly likely differentially expressed genes in a latent biological process, we then can link the latent variable with a real biological pathway. In recent years, probabilistic topic models have spawn many similar works on genomic data, noticeably in population genetics (Pritchard et al., 2000), chemogenomic profiling (Flaherty et al., 2005) and microarray data (Rogers et al., 2005; Bicego et al., 2012; Yu et al., 2014). However, most of the previous works of probabilistic topic models on microarray data either have limited size of samples, or probabilistic topic models are used merely for their clustering ability. The versatility of probabilistic topic models has not been fully assessed.

We proposed a probabilistic topic model that was tailored to the structure of a dataset and applied the model to a large toxicogenomics database recently made publicly available. This so-called asymmetric author-topic model (ATT model) combines author-topic model (Rosen-Zvi et al., 2004) with asymmetric prior (Wallach et al., 2009). In Section Materials and Methods, we outlined our data, the proposed model and its application to toxicogenomic data. In Section Results, we presented the analysis results. Analyses were done with MALLET (McCallum, 2002) that contains the option for asymmetric prior distributions.

## Materials and methods

### Toxicogenomic data

The Japanese Toxicogenomics Project (Uehara et al., 2010; Chen et al., 2012) is a 10-year collaborative project involving two Japanese government institutes and 18 private companies (Igarashi et al., 2015). The project produced a comprehensive gene expression database, called Open TG-GATEs for the effects of 170 compounds (drugs) on liver and kidney as primary target organs in both *in vivo* and *in vitro* experiments. Specifically, in the *in vivo* experiment, animals are treated at three different doses (low, middle, and high) of drugs once every day for four different treatment durations (3, 7, 14, and 28 days). In addition, control animals are concurrent with all the 12 combinations of doses and durations. More details on the animals and experimental design have been described previously (Uehara et al., 2011). Microarray based gene expression data were generated using the GeneChip® Rat Genome 230 2.0 Arrays (Affymetrix, Santa Clara, CA, USA) that contains 31,042 probe sets. The data used in this study is obtained from the Annual International Conference on Critical Assessment of Massive Data Analysis (CAMDA)^1^ 2013 (http://dokuwiki.bioinf.jku.at/doku.php/tgp_prepro). In this paper, only the data from *in vivo* repeated dose experiment was used.

### Data preprocessing

Similar to others (Rogers et al., 2005; Bicego et al., 2010; Yu et al., 2014), our first step of analysis was to obtain a “document-word” matrix for gene expression data to apply topic model. Instead of the sample-gene expression matrix used in others' works, we created treatment-fold change matrix for our studies. This was due to the fact that TG-GATEs has multiple treated samples for one treatment (a unique drug-time-dose combination) along with controlled group. Therefore, we were able to apply a more refined treatment-fold change matrix as our inputs. Here, all fold change values of gene expressions between treated and control samples were calculated and used as the value of elements of the matrix. Genes with absolute fold change greater than 1.5 were considered as differentially expressed genes (DEGs) and set the fold change values zeros for the non-DEG. The final product is a treatment-fold change matrix where each column represents a treatment and each row represents a gene.

### Probabilistic topic models and their applications

#### Latent dirichlet allocation in microarray data

The fundamental concept of probabilistic topic modeling is the assumption of the existence of latent variables. In Latent Dirichlet Allocation (LDA) (Blei et al., 2003), the latent variables are referred as “topics” and words in documents are chosen based on what topics the document are related to. “Topics” then stands for groups of words that are likely to co-occur in a document. Similar to the previous studies (Bicego et al., 2010; Yu et al., 2014), we referred latent variables in toxicogenomics as “latent biological process” and words in documents were replaced by genes. The elements of document-word matrix, which usually are frequencies of occurrences of words in text mining, were transformed to the fold change values in our treatment-gene matrix. Hence, the latent biological processes represent the groups of genes that are significantly co-expressed (or often have high fold change values within groups). Unlike (Rogers et al., 2005) which alters the original assumption of LDA model, we utilized the original assumption of LDA and this enabled us to implement our models via existing resources of LDA (i.e., MALLET, the open-source software used in our analysis). Therefore, similar to LDA, the model inferences were primarily focused on two probability distributions. In the context of TG-GATEs data, the probability distribution of latent biological processes for each treatment is *P*(*Z*|*Tr*), where *Z* is defined as latent process assignment while *Tr* is defined as treatment to describe biological processes that are activated in a specific treatment. Meanwhile, the probability distribution of gene for each latent biological process is *P*(*Ge*|*Z*), where *Ge* is defined as genes that are DEGs from which we are able to associate the latent process to biological pathways. The ability of linking latent process to biological pathway is a definite advantage over other clustering algorithms and we explored its applications in Section Functional Annotation and Similarity Ranking.

#### Asymmetric author-topic model

Although LDA could be used for treatment-centric analysis, it doesn't take many unique features of the TG-GATEs data into account. In addition to examine the treatment-centric view, drug-centric and/or time-dose-centric analysis were another important component of this study. The author-topic model (Rosen-Zvi et al., 2004) is a proper methodology to incorporate other aspects of data into model construction. Authorship in author-topic model can be seen as a regrouping of all the documents. While both models are essentially identical, author-topic model groups documents together and give LDA model an author-oriented view for inferences. In other words, once the regrouping is done, the whole process can be seen as an LDA model again. For TG-GATEs data, treatment is defined as a unique drug-time-dose combination, thus we can regroup treatments based on their drug or time-dose to provide a drug-centric or a time-dose-wise analysis. The inferences on models are the same except treatment is replaced by either drug or time-dose. Furthermore, *P*(*Z*|*Tr*) is replaced by *P*(*Z*|*Dr*) (*Dr* stands for Drug) and *P*(*Z*|*DoTi*) (*DoTi* stands for time-dose) respectively. Table 1 summarizes the total number of individuals in each setting.

**Table 1:** **Summary of different feature specifications of asymmetric author-topic model**.

**Model no**.	**Feature**	**Number of individuals**	**Outputs**
1	Treatment	1554	*P*(*Ge*|*Z*), *P*(*Z*|*Tr*)
2	Drug	131	*P*(*Ge*|*Z*), *P*(*Z*|*Dr*)
3	Time-dose	12	*P*(*Ge*|*Z*), *P*(*Z*|*DoTi*)

As Wallach et al. (2009) pointed out, asymmetric prior on the probability distribution of topic for a document substantially increases the robustness of LDA, yet only adds negligible model complexity and computational cost. Therefore, we further improved author-topic model by introducing an asymmetric prior. The asymmetry of priors can be easily achieved since the chosen software MALLET has a build-in option in the command. More information about MALLET can be found on their website (http://mallet.cs.umass.edu/).

### Functional annotation and similarity ranking

One essential aspect of any clustering algorithm is to organize individuals into their respective clusters. However, the clusters often are difficult to interpret. Through asymmetric author-topic model, individuals are clustered to multiple latent biological processes based on the probability distribution *P*(*Z*|*Tr*) (or *P*(*Z*|*Dr*), *P*(*Z*|*DoTi*)). For each latent biological process, probability distribution *P*(*Ge*|*Z*) controls how likely each gene is differentially expressed (i.e., a DEG). According to our results, there are often fewer than 200 genes (out of 31,042 total genes) that have positive probability in each latent biological process while other genes have probability of zeros. We then annotate the found list of DEGs in each latent biological process through online database DAVID (Huang Da et al., 2009). Consequently, every feature (i.e., treatment, drug, or time-dose) in the database is automatically connected to annotated biological pathways. The ability of our proposed model to link from the latent biological processes to functional annotation, such as real biological pathways, is a significant advantage over other existing methods.

Another application of author-topic model is to find most similar feature to a given one. We can quantitatively measure the similarity between a pair of features by calculating the symmetric Kullback–Leibler divergence (sKL) (Rosen-Zvi et al., 2004) between a pair of *P*(*Z*|*Tr*) (or *P*(*Z*|*Dr*), *P*(*Z*|*DoTi*)). For instance, by finding the sKL between *P*(*Z*|*Dr*_1_) and *P*(*Z*|*Dr*_2_), we can tell how similar Drug 1 and Drug 2 is (i.e., a low sKL score indicates that two drugs exhibit similar topic distributions). Given a drug, our model is able to recommend a list of drugs ranked by the similarity score sKL. Due to (1) the similarity is based on *P*(*Z*|*Dr*), the probability of latent biological processes given drugs, and (2) all the latent biological processes are able to annotated to biological pathways, we know which drugs are similar as well as exactly which pathways link them together.

## Results

### Model selection

We run all three of our models on MALLET, whose model inference is based on Gibbs sampling algorithm. One common concern using Gibbs sampling is the convergence of the model. Generally, convergence of the model is monitored via tracking the probability of the likelihood function after burn-in. After the likelihood probability stabilizes, we can deem convergence to be adequate. We run 3000 iterations for all models and observe stability after about 1500 iterations. We also perform sensitivity analyses for major parameters, including number of latent biological processes, and the initial values for hyperpriors. Hyperpriors are usually not big factors in the model as they are constantly revised during rounds of Gibbs sampling inference. On the other hand, the number of latent biological processes is important. While there is no way to know how many biological processes are involved in the whole database, we can estimate the number based on perplexity performance (Blei et al., 2003). In addition, asymmetric topic models have been shown to be robust to variations in the number of topics (Wallach et al., 2009). All the parameters are chosen based on 10-fold cross-validation. For model 1 (treatment), the number of latent biological processes is 200. For models 2 and 3 (drug and time-dose) the number of latent biological processes is 100.

### Application on glutathione depletion

One proven application of TGP database is detection of glutathione depletion (Uehara et al., 2010). Taking well-known hepatotoxin acetaminophen as an example, it was reported that glutathione metabolism was related to acetaminophen-induced hepatotoxicity and the mechanisms that underline such liver injury (Agarwal et al., 2011; Ben-Shachar et al., 2012). For instance, James et al. (2003) pointed out that acetaminophen could induce potentially fatal, hepatic centrilobular necrosis when taken in overdose, since the amount of active metabolite overwhelmed the detoxification capacity of intracellular glutathione. Among our proposed models, model 1 gives us a treatment-centric view of the TGP database. Table 2 shows *P*(*Z*|*Tr*) from model 1 that represents the most likely latent biological processes that encode biological phenomena associated with acetaminophen. Latent process 161 is identified in 8 out of 12 time-dose combinations for acetaminophen, as early as the 3-day treatment with the middle dose of 600 mg. Furthermore, the list of most probable DEGs for latent process 161 is extracted from *P*(*Ge*|*Z*) and functionally annotated by online database DAVID. As seen on Table 3, glutathione metabolism pathway is significantly identified in the KEGG database, which is consistent with the previous findings.

**Table 2:** **The probability of latent biological processes for acetaminophen under model 1**.

**Treatment index**	**Dose**	**Time (Days)**	**Top ranked latent biological processes**					
			**1**	**Probability**	**2**	**Probability**	**3**	**Probability**
**ACETAMINOPHEN**								
36	Low	3	2	0.149	36	0.124	181	0.122
37	Middle	3	**161**	0.279	111	0.168	116	0.098
38	High	3	**161**	0.139	39	0.1	169	0.1
39	Low	7	68	0.305	162	0.211	69	0.165
40	Middle	7	**161**	0.366	149	0.12	57	0.079
41	High	7	**161**	0.275	27	0.08	39	0.066
42	Low	14	69	0.153	134	0.138	63	0.138
43	Middle	14	**161**	0.342	128	0.104	37	0.098
44	High	14	**161**	0.274	113	0.082	128	0.074
45	Low	28	69	0.175	96	0.175	160	0.153
46	Middle	28	**161**	0.278	96	0.152	14	0.085
47	High	28	**161**	0.366	197	0.091	164	0.07

*Only top three topics for each different treatment (drug-dose-time) are shown. For full table, see Supplementary 1*.

*Topic 161 (in bold) is significantly associated with glutathione metabolism*.

**Table 3:** **Functional annotation of KEGG pathways on latent biological process 161 under model 1**.

**Term**	**Count**	**FDR**	***P*-value**	**Genes**
rno00480:Glutathione metabolism	8	1.55E-05	1.65E-08	GPX2, GSR, GCLC, G6PD, GSTA5, GCLM, GSTP1, MGST2
rno00980:Metabolism of xenobiotics by cytochrome P450	7	0.00142	1.51E-06	GSTA5, ADH4, UGT2B1, EPHX1, CYP3A9, GSTP1, MGST2
rno00982:Drug metabolism	7	0.00420	4.47E-06	GSTA5, ADH4, UGT2B1, AOX1, CYP3A9, GSTP1, MGST2

*Functional annotation is done on online database David. Only the top 3 annotated of KEGG pathway terms are shown here. For full table, see Supplementary 2*.

In model 2, the drug-centric view of the TGP database, we observe similar results. Again, the most likely active latent process for acetaminophen is latent process 92 (Table 4) and it is once again significantly identified as glutathione metabolism pathway in the KEGG database (Table 5). In addition, by simply searching the drugs that also have No. 92 among the top ranked latent processes, we find that bromobenzene, chlormezanone, coumarin, methimazole, and ticlopidine strongly link with glutathione metabolism pathway (Table 4), and hence presumably become causes of glutathione depletion. Such hepatotoxicity associated with these 6 drugs through the glutathione metabolism pathway is well supported in other papers (Jollow et al., 1974; Thor et al., 1979; Wright et al., 1996; Mizutani et al., 2000; Uehara et al., 2010; Shimizu et al., 2011). Overall, our results indicate that the construction of our proposed model indeed matches with the well-known biological processes and hence the model is able to detect potential treatments or drugs that cause glutathione depletion.

**Table 4:** **The probability of latent biological processes for acetaminophen, bromobenzene, chlormezanone, coumarin, methimazole, and ticlopidine under model 2**.

**Drug Index**	**Drug**	**Top ranked latent biological processes**					
		**1**	**Probability**	**2**	**Probability**	**3**	**Probability**
3	Acetaminophen	**92**	0.201	17	0.190	1	0.118
16	Bromobenzene	**92**	0.318	1	0.138	17	0.125
27	Chlormezanone	9	0.341	**92**	0.192	1	0.128
37	Coumarin	98	0.293	**92**	0.193	1	0.142
81	Methimazole	**92**	0.211	21	0.185	32	0.143
123	Ticlopidine	9	0.248	**92**	0.093	1	0.089

*Again, only top three latent processes for each drug are shown. For full table, see Supplementary 3*.

*Topic 92 (in bold) is significantly associated with glutathione metabolism*.

**Table 5:** **Functional annotation of KEGG pathways on latent biological process 92 under model 2**.

**Term**	**Count**	**FDR**	***P*-value**	**Genes**
rno00480:Glutathione metabolism	11	5.67E-07	5.18E-10	GSTM1, GPX2, GSR, GCLC, GSTM4, G6PD, GSTA5, GSTT1, GCLM, GSTP1, GSTM7, MGST2
rno00980:Metabolism of xenobiotics by cytochrome P450	9	9.31E-04	8.51E-07	GSTM1, GSTM4, GSTA5, ADH4, UGT2B1, EPHX1, GSTT1, GSTP1, GSTM7, MGST2
rno00982:Drug metabolism	9	0.00384	3.51E-06	GSTM1, GSTM4, GSTA5, ADH4, UGT2B1, AOX1, GSTT1, GSTP1, GSTM7, MGST2

*Functional annotation is done on online database David. Only the top 3 annotated of KEGG pathway terms are shown here. For full table, see Supplementary 4*.

### Application of drug similarity and potential reduction of animal use

Through sKL score (described in Section Functional Annotation and Similarity Ranking), functional similarity of drugs can be explored. As an example, we can obtain the most functionally similar drugs to acetaminophen as shown in Table 6. The drugs that have smaller sKL score with acetaminophen (i.e., a pair-wise score) will exhibit most similar latent biological processes. We can observe that bromobenzene and coumarin, which linked through glutathione depletion pathway, are on the list.

**Table 6:** **Most similar drugs to acetaminophen based on sKL scores**.

**Drug name**	**sKL score**
Bromobenzene	3.04238
Phenacetin	4.47157
Bucetin	4.51243
Cimetidine	5.46445
Disopyramide	5.85482
Cephalothin	5.89109
Papaverine	5.92761
Erythromycin ethylsuccinate	5.92976
Coumarin	6.03134
Nitrofurantoin	6.03479

*The smaller the sKL is, the more similar two drugs are. Only top 10 ranked drugs are shown here. For full table, see Supplementary 5*.

Another application of sKL score is to be used as potential evidence of reduction of animal use. Reducing, replacing and refining animal use (3Rs) has been increasingly a goal in toxicogenomics (Russell et al., 1959; Workman et al., 2010). While dose level and time-point are expected to be important, there is generally no easy way to determine which treatment is ignorable for a given drug. sKL scores measure the similarity between a pair of treatments. The idea is to see if either dose or time in treatments of a drug does not play a significant role to affect sKL score. If one of them is not significant to sKL score, then there exists the potential to reduce the number of treatments without compromising study goals. Similar to multivariate analysis of variance (MANOVA), the importance of dose and time can be attained with generalized linear models on sKL scores as the following:

$$\begin{array}{l} sKL=\beta_{1}X_{Dose}+\beta_{2}X_{Time},\\ sKL=\beta_{1}X_{Dose},or\\ sKL=\beta_{1}X_{Time} \end{array}$$

Here, *X_Dose_* is defined as a categorical variable that includes six different dose pairs (i.e., Low-Low, Low-Middle, Low-High, Middle-Middle, Middle-High, and High-High). *X_Time_* is defined as a continuous non-negative variable that represents the difference between two time-points. By fitting the generalized linear model using various common model criteria (e.g., adjusted R-square, AIC, and BIC), we can compare dose and/or time significance regarding to sKL score. A level of feature that has no significant impact on sKL score can be potentially reduced. While only having 12 individuals, model 3 can be used to detect the overall significance of dose and time. Unsurprisingly, dose and time generally are both significant to sKL score as seen in Table 7. It is naïve to think we can remove any treatment regardless which drug is been tested, yet there might be specific drugs that fit our assumption. As examples, we chose acetaminophen, coumarin, and benzbromarone to be tested in the generalized linear models. Among all, only benzbromarone consistently demonstrate the superiority of dose only model under all three model criteria. Therefore, it is possible to combine time-points for treatments of benzbromarone due to the insignificance of time regarding to sKL score.

**Table 7:** **Generalized linear models for sKL scores under three (Adjusted R-square, AIC, and BIC) criteria, with best outcomes bolded**.

**GLMs**	**Adjusted *R*-square**			**AIC**			**BIC**		
	**D and T**	**Dose**	**Time**	**D and T**	**Dose**	**Time**	**D and T**	**Dose**	**Time**
Model 3	**0.456**	0.437	0.076	**82.703**	93.771	117.212	**98.030**	106.909	121.591
Acetaminophen	**0.559**	0.453	0.051	**204.660**	216.462	246.815	**219.988**	229.600	251.194
Coumarin	**0.592**	0.583	0.016	258.487	**257.649**	296.490	273.814	**270.786**	300.869
Benzbromarone	0.813	**0.816**	0.004	225.281	**223.221**	340.736	240.609	**236.359**	345.115

## Discussion

Our proposed asymmetric author-topic model is useful in the large-scale genomics data set analysis because of their ability to handle large numbers of potentially interrelated variables, and because of their ability to discern statistical relationships between drugs and their inner pathways. In this paper, we first give our rationale on why a probabilistic topic model is suitable for genomic profiling expression, such as the Japanese Toxicogenomics Project database. We have demonstrated that our asymmetric author-topic model can be implemented to explore hidden relationships among different features (treatment, drug, and time-dose) and genes through latent biological processes. The straightforward data preprocessing makes the transition of data format manageable and easy to expand. In fact, the same principle of data preprocessing can also be applied to next-generation sequencing (NGS) technology since microarray expression intensity can be simply replaced by read counts in NGS (Yu et al., 2014). Since our model enhances the traditional probabilistic topic modeling approach without altering the core assumptions, our framework can be easily adapted for new probabilistic topic model. For example, if we have labels or classes attached to each treatment, we can again enhanced supervised topic models (Blei and McAuliffe, 2007) with asymmetric priors and applied the model on database with same feature-centric analysis capacity. Because of the popularity of probabilistic topic modeling, there are many existing and well-built software packages ready to be used, including MALLET. Therefore, the implementation of newer probability topic models should also be straightforward in the future. Moreover, other models can also potentially improve some of the limitation our model has. Although changing a continuous value (i.e., fold change values) into a discrete value (i.e., counts) has been done before (Flaherty et al., 2005), this process ultimately decrease the precision of the data. Models like Gaussian mixture model that supports continuous outcome will eliminate the need of altering data. Another limitation of our model is the need to determine the number of latent biological processes in advanced. While the perplexity analysis ensures a relatively proper number of latent processes were chosen initially, finding an optimal number of latent processes is still difficult and costly. Many nonparametric Bayesian models has been developed, including Hierarchical Dirichlet Processes (Teh et al., 2006), and Hierarchical Pachinko Allocation (Mimno et al., 2007), and the number of latent processes is automatically determined within the algorithm.

One definite advantage of asymmetric author-topic model is the ability to connect the latent biological processes with functional annotation. By connecting our finding with KEGG pathways via DAVID, we further increase the interpretability of latent biological processes. Therefore, we are able to browse and interact with TGP data through meaningful and interpretable biological pathway (i.e., glutathione metabolism). Regarding the application on glutathione depletion, acetaminophen is a well-known drug that can potentially cause fatal liver injury due to an overdose. Through our approach, we identify that the alteration of glutathione metabolism at even the middle dose (600 mg) of acetaminophen as early as treatment day three. The conclusion of linkages among pathway glutathione metabolism, acetaminophen, and other 5 drugs are found and confirmed in other papers. This demonstrates the possibility of finding existing or new pathway-like annotation through our proposed model, and the ability to cluster drugs with similar mechanisms of action. It is possible to even predict potential pathways for a new drug by estimating the probability distribution of latent biological processes under this framework. Our model also has the capability to adapt analysis that put focus on different features of data. We show how to identify the dominant factor in dose and time combinations in our second application through generalized linear model. As animal reduction in experiment becomes a global trend, the outcome of similarity of time-dose combination is a viable approach to reducing animals needed for future study. Overall, the asymmetric author-topic model has demonstrated potential to be an accessible and flexible approach for finding hidden patterns in large toxicogenomic data.

### Conflict of interest statement

The authors declare that the research was conducted in the absence of any commercial or financial relationships that could be construed as a potential conflict of interest.

## Footnotes

^1^Download preprocessed TGP data - CAMDA 2013 [Online]. Available online at: http://dokuwiki.bioinf.jku.at/doku.php/tgp_prepro [Accessed 04/08 2014].

## References

[B1] Afshari C. A. Hamadeh H. K. Bushel P. R. (2011). The evolution of bioinformatics in toxicology: advancing toxicogenomics. Toxicol. Sci. 120(Suppl. 1), S225–S237. 10.1093/toxsci/kfq373 21177775 PMC3145387

[B2] Agarwal R. Macmillan-Crow L. A. Rafferty T. M. Saba H. Roberts D. W. Fifer E. K.. (2011). Acetaminophen-induced hepatotoxicity in mice occurs with inhibition of activity and nitration of mitochondrial manganese superoxide dismutase. J. Pharmacol. Exp. Ther. 337, 110–116. 10.1124/jpet.110.176321 21205919 PMC3063736

[B3] Ben-Shachar R. Chen Y. Luo S. Hartman C. Reed M. Nijhout H. F. (2012). The biochemistry of acetaminophen hepatotoxicity and rescue: a mathematical model. Theor. Biol. Med. Model. 9:55. 10.1186/1742-4682-9-55 23249634 PMC3576299

[B4] Bicego M. Lovato P. Ferrarini A. Delledonne M. (2010). Biclustering of expression microarray data with topic models, in Pattern Recognition (ICPR), 2010 20th International Conference on (Istanbul: IEEE), 2728–2731.

[B5] Bicego M. Lovato P. Perina A. Fasoli M. Delledonne M. Pezzotti M.. (2012). Investigating topic models' capabilities in expression microarray data classification. IEEE/ACM Trans. Comput. Biol. Bioinform. 9, 1831–1836. 10.1109/TCBB.2012.121 23221091

[B6] Blei D. M. McAuliffe J. D. (2007). Supervised topic models, in NIPS (Vancouver, BC), 121–128.

[B7] Blei D. M. Ng A. Y. Jordan M. I. (2003). Latent dirichlet allocation. J. Mach. Learn. Res. 3, 993–1022.

[B8] Chen M. Zhang M. Borlak J. Tong W. (2012). A decade of toxicogenomic research and its contribution to toxicological science. Toxicol. Sci. 130, 217–228. 10.1093/toxsci/kfs223 22790972

[B9] Derisi J. Penland L. Brown P. O. Bittner M. L. Meltzer P. S. Ray M.. (1996). Use of a cDNA microarray to analyse gene expression patterns in human cancer. Nat. Genet. 14, 457–460. 10.1038/ng1296-457 8944026

[B10] Flaherty P. Giaever G. Kumm J. Jordan M. I. Arkin A. P. (2005). A latent variable model for chemogenomic profiling. Bioinformatics 21, 3286–3293. 10.1093/bioinformatics/bti515 15919724

[B11] Fu L. Medico E. (2007). FLAME, a novel fuzzy clustering method for the analysis of DNA microarray data. BMC Bioinformatics 8:3. 10.1186/1471-2105-8-3 17204155 PMC1774579

[B12] Hofmann T. (1999). Probabilistic latent semantic indexing, in Proceedings of the 22nd Annual International ACM SIGIR Conference on Research and Development in Information Retrieval (Berkeley, CA: ACM), 50–57.

[B13] Huang Da W. Sherman B. T. Lempicki R. A. (2009). Systematic and integrative analysis of large gene lists using DAVID bioinformatics resources. Nat. Protoc. 4, 44–57. 10.1038/nprot.2008.211 19131956

[B14] Igarashi Y. Nakatsu N. Yamashita T. Ono A. Ohno Y. Urushidani T.. (2015). Open TG-GATEs: a large-scale toxicogenomics database. Nucleic Acids Res. 43, D921–D927. 10.1093/nar/gku955 25313160 PMC4384023

[B15] James L. P. Mayeux P. R. Hinson J. A. (2003). Acetaminophen-induced hepatotoxicity. Drug Metab. Dispos. 31, 1499–1506. 10.1124/dmd.31.12.1499 14625346

[B16] Jollow D. Mitchell J. Zampaglione N. A. Gillette J. (1974). Bromobenzene-induced liver necrosis. Protective role of glutathione and evidence for 3, 4-bromobenzene oxide as the hepatotoxic metabolite. Pharmacology 11, 151–169. 10.1159/000136485 4831804

[B17] McCallum A. K. (2002). MALLET: A Machine Learning for Language Toolkit. Available online at: http://mallet.cs.umass.edu

[B18] Mimno D. Li W. McCallum A. (2007). Mixtures of hierarchical topics with pachinko allocation, in Proceedings of the 24th International Conference on Machine Learning (Corvallis, OR: ACM), 633–640.

[B19] Mizutani T. Yoshida K. Murakami M. Shirai M. Kawazoe S. (2000). Evidence for the involvement of N-methylthiourea, a ring cleavage metabolite, in the hepatotoxicity of methimazole in glutathione-depleted mice: structure-toxicity and metabolic studies. Chem. Res. Toxicol. 13, 170–176. 10.1021/tx990155o 10725113

[B20] Nuwaysir E. F. Bittner M. Trent J. Barrett J. C. Afshari C. A. (1999). Microarrays and toxicology: the advent of toxicogenomics. Mol. Carcinog. 24, 153–159. 10204799 10.1002/(sici)1098-2744(199903)24:3<153::aid-mc1>3.0.co;2-p

[B21] Pal N. R. Bezdek J. C. (1995). On cluster validity for the fuzzy c-means model. IEEE Trans. Fuzzy Syst. 3, 370–379 10.1109/91.413225

[B22] Pritchard J. K. Stephens M. Donnelly P. (2000). Inference of population structure using multilocus genotype data. Genetics 155, 945–959. 10835412 10.1093/genetics/155.2.945 PMC1461096

[B23] Rogers S. Girolami M. Campbell C. Breitling R. (2005). The latent process decomposition of cDNA microarray data sets. IEEE/ACM Trans. Comput. Biol. Bioinform. 2, 143–156. 10.1109/TCBB.2005.29 17044179

[B24] Rosen-Zvi M. Griffiths T. Steyvers M. Smyth P. (2004). The author-topic model for authors and documents, in Proceedings of the 20th Conference on Uncertainty in Artificial Intelligence (Banff, AB: AUAI Press), 487–494.

[B25] Russell W. M. S. Burch R. L. Hume C. W. (1959). The Principles of Humane Experimental Technique. St. Albans: Universities Federation for Animal Welfare (UFAW).

[B26] Schena M. Shalon D. Davis R. W. Brown P. O. (1995). Quantitative monitoring of gene expression patterns with a complementary DNA microarray. Science 270, 467–470. 10.1126/science.270.5235.467 7569999

[B27] Shimizu S. Atsumi R. Nakazawa T. Izumi T. Sudo K. Okazaki O.. (2011). Ticlopidine-induced hepatotoxicity in a GSH-depleted rat model. Arch. Toxicol. 85, 347–353. 10.1007/s00204-010-0594-9 20871981

[B28] Teh Y. W. Jordan M. I. Beal M. J. Blei D. M. (2006). Hierarchical dirichlet processes. J. Am. Stat. Assoc. 101, 1566–1581 10.1198/016214506000000302

[B29] Thor H. Moldéus P. Orrenius S. (1979). Metabolic activation and hepatotoxicity: effect of cysteine, N-acetylcysteine, and methionine on glutathione biosynthesis and bromobenzene toxicity in isolated rat hepatocytes. Arch. Biochem. Biophys. 192, 405–413. 10.1016/0003-9861(79)90109-7 434834

[B30] Uehara T. Minowa Y. Morikawa Y. Kondo C. Maruyama T. Kato I.. (2011). Prediction model of potential hepatocarcinogenicity of rat hepatocarcinogens using a large-scale toxicogenomics database. Toxicol. Appl. Pharmacol. 255, 297–306. 10.1016/j.taap.2011.07.001 21784091

[B31] Uehara T. Ono A. Maruyama T. Kato I. Yamada H. Ohno Y.. (2010). The Japanese toxicogenomics project: application of toxicogenomics. Mol. Nutr. Food Res. 54, 218–227. 10.1002/mnfr.200900169 20041446

[B32] Wallach H. M. Mimno D. M. McCallum A. (2009). Rethinking LDA: why priors matter, in NIPS (Vancouver, CA), 1973–1981.

[B33] Workman P. Aboagye E. Balkwill F. Balmain A. Bruder G. Chaplin D.. (2010). Guidelines for the welfare and use of animals in cancer research. Br. J. Cancer 102, 1555–1577. 10.1038/sj.bjc.6605642 20502460 PMC2883160

[B34] Wright R. O. Perry H. E. Woolf A. D. Shannon M. W. (1996). Hemolysis after acetaminophen overdose in a patient with glucose-6-phosphate dehydrogenase deficiency. Clin. Toxicol. 34, 731–734. 10.3109/15563659609013837 8941205

[B35] Yu K. Gong B. Lee M. Liu Z. Xu J. Perkins R.. (2014). Discovering functional modules by topic modeling RNA-Seq based toxicogenomic data. Chem. Res. Toxicol. 27, 1528–1536. 10.1021/tx500148n 25083553

