# Supplementary material for: Asymmetric author-topic model for knowledge discovering of big data in toxicogenomics
Source: Front Pharmacol. 2015 Apr 20;6:81. doi: 10.3389/fphar.2015.00081 (PMC4403303; doi:10.3389/fphar.2015.00081)
Supplement: Supplementary file 1 [file DataSheet1.DOCX]

***Supplementary Material***

**Discover the hidden biological pathways in large toxicogenomic data via probabilistic topic modeling**

**Ming-Hua Chung ^1#^, Yuping Wang^2#^, Hailin Tang^2^, Wen Zou^2^, John Basinger^2^, Xiaowei Xu^3^*, and Weida Tong^2^***

^1^Department of Mathematical Sciences, University of Arkansas, Fayetteville, AR, USA ^2^Division of Bioinformatics and Biostatistics, National Center for Toxicological Research, US Food and Drug Administration, Jefferson, AR, USA

^3^Department of Information Science, University of Arkansas at Little Rock, Little Rock, AR, USA

**^#:^** These authors contributed equally.

*** Correspondence:** Xiaowei Xu, Department of Information Science, 2801 S University Avenue EIT 550, Little Rock, AR, 72204, USA.

[xwxu@ualr.edu](mailto:xwxu@ualr.edu)

Weida Tong, Division of Bioinformatics and Biostatistics, National Center for Toxicological Research, US Food and Drug Administration, 3900 NCTR Rd., Jefferson, AR, 72079, USA.

[Weida.Tong@fda.hhs.gov](mailto:Weida.Tong@fda.hhs.gov)

1. **Supplementary Tables**

Here we listed some of the tables used in the paper in their entirety.

## Supplementary Tables

**Supplementary Table 1. The probability of latent biological processes for acetaminophen under model 1.** The table shows all 200 probability distributions $P(Z|Tr)$ of latent biological processes for all 1554 treatments (drug-time-dose combinations). Each row represents a unique treatment and probabilities $P(Z|Tr)$ were listed sequentially from the largest to the smallest. Both No. for treatment and latent process were started from 0.

**Supplementary Table 2. Functional annotation of KEGG pathways on latent biological process 161 under model 1.** This file was directed exported out of “Functional Annotation Chart” from online database David (<http://david.abcc.ncifcrf.gov/home.jsp>).

**Supplementary Table 3. The probability of latent biological processes for acetaminophen, bromobenzene, chlormezanone, coumarin, methimazole, and ticlopidine under model 2.** The table shows all 200 probability distributions $P(Z|Dr)$ of latent biological processes for all 131 drugs. Each row represents a unique drug and probabilities $P(Z|Dr)$ were listed sequentially from the largest to the smallest. Both No. for drug and latent process were started from 0.

**Supplementary Table 4. Functional annotation of KEGG pathways on latent biological process 92 under model 2.** This file was directed exported out of “Functional Annotation Chart” from online database David (<http://david.abcc.ncifcrf.gov/home.jsp>).

**Supplementary Table 5. Most similar drugs to acetaminophen based on sKL scores.** The smaller the sKL is, the more similar two drugs are. The table had been sorted to show the most similar drugs to acetaminophen. No. for drug was started from 0.

**Supplementary Table 6. The preprocessed data for treatment-centric analysis.** Following section 2.2, this is preprocessed dataset for treatment-centric analysis (Model 1). The format of the data is required by MALLET. Each row represents a treatment (drug-dose-time combination). First column indicates the treatment name. Second column indicates class, which is unused and set to be “A” deliberately. From third column and on, significantly differentially expressed genes (DEGs) are listed according to their fold change values. Due to the fact that the number of appearances can only be associated with positive integers, all fold changed values are rounded to positive integers. For instance, in treatment “WY-14643_4day_Low”, gene “Dhrs4” has fold change values rounded to 2, and hence appeared twice in row 1. Eventually, this dataset is loaded by MALLET and MALLET will automatically generate treatment-fold change matrix (section 2.2) and perform analysis based on the matrix.
